# Imbalance in excitation and inhibition in sgACC is associated with depression in dementia with Lewy bodies

**DOI:** 10.1038/s41398-026-04224-x

**Published:** 2026-07-03

**Authors:** Lina Gliaudelytė, Steven P. Rushton, Alan J. Thomas, Christopher M. Morris

**Affiliations:** 1 Alzheimer’s Society Doctoral Training Centre, Translational and Clinical Research Institute, Edwardson Building, Faculty of Medical Sciences, Newcastle University, Newcastle upon Tyne, Tyne And Wear NE4 5PL UK; 2 NIHR Newcastle Biomedical Research Centre (BRC), Biomedical Research Building, Faculty of Medical Sciences, Campus for Ageing and Vitality, Newcastle upon Tyne, Tyne And Wear NE4 5PL UK; 3 School of Natural and Environmental Sciences, Agriculture Building, Newcastle University, Newcastle upon Tyne, Tyne And Wear NE1 7RU UK; 4 Queen Elizabeth Hospital, Queen Elizabeth Avenue, Gateshead, Tyne and Wear NE9 6SX UK; 5 Newcastle Brain Tissue Resource, Translational and Clinical Research Institute, Edwardson Building, Faculty of Medical Sciences, Newcastle University, Newcastle upon Tyne, Tyne And Wear NE4 5PL UK

**Keywords:** Molecular neuroscience, Diseases

## Abstract

Depression in dementia with Lewy bodies (DLB) is a common neuropsychiatric symptom associated with reduced quality in life. Structural, functional and neurochemical abnormalities in glutamatergic and GABAergic neurotransmission are implicated in the pathophysiology of depression, showing changes in regions involved in emotional processing, including the subgenual cingulate cortex (sgACC), which shows pathological changes in DLB. Using post-mortem tissue from DLB patients and controls, we assessed synaptic and neurochemical changes within the sgACC in relation to depression in DLB. We identified a reduction of layer V GABAergic neurones in depressed DLB cases, potentially indicating reduced inhibition of layer V pyramidal neurons, leading to altered excitation. High-resolution confocal imaging demonstrated a significantly increased volume of presynaptic glutamatergic synapses containing phosphorylated α-synuclein (s129) in DLB cases, and specifically in depressed DLB cases, potentially as a compensatory response to the accumulation of pathological s129. GABAergic synapses containing s129 were enlarged in both DLB groups showing no depression specific changes. Selective reductions in glutamatergic and GABAergic receptors were seen in depressed DLB cases, suggesting a role in the pathophysiology of depression in DLB, that may prove amenable to therapy with fast-acting antidepressants. Interactions with serotonergic and dopaminergic innervation were observed, where preserved 5HT3B receptor and calbindin characterised non-depressed DLB patients. In depressed DLB cases, this may lead to reduced inhibition of lower layer pyramidal neurones due to reduced dopaminergic coupling and enhanced excitatory activity within the cingulate. Overall, our findings suggest altered excitatory and inhibitory neurotransmission may contribute to the development of depression in DLB.

## Introduction

Dementia with Lewy bodies (DLB) accounts for about 20% of cases of dementia reaching autopsy [1, 2]. DLB is diagnosed using four core clinical features (cognitive fluctuations, parkinsonism, recurrent complex visual hallucinations and rapid eye movement sleep behaviour disorder (RBD)) together with indicative biomarkers (polysomnographic evidence of RBD, dopaminergic imaging, MIBG (metaiodobenzylguanidine) imaging) [3]. Whilst not a diagnostic feature, depression is a common neuropsychiatric symptom in DLB, observed in over half of patients [4], which contributes to faster cognitive decline and reduced quality of life [5, 6]. The causes of these different clinical symptoms in DLB are essentially unknown, although may ultimately relate to the presence of α-synuclein protein containing pathology within widespread cortical and subcortical brain regions [3]. In previous studies we have observed significant changes in dopaminergic neurotransmission associated with depression in DLB [7, 8]. Whilst dopamine changes in DLB may reflect some of the pathophysiology associated with depression in DLB, the complex aetiology of depression may involve additional neurotransmitter systems.

Increasing evidence supports an impairment of glutamatergic and GABAergic neurotransmission in the pathophysiology of depression [9, 10]. Clinical studies demonstrate abnormal glutamatergic transmission in limbic and cortical areas involved in emotional processing in depressed individuals [10, 11]. Abnormal glutamatergic signalling has also been observed in post-mortem and MRI studies in patients with major depressive disorder (MDD) [12], contributing to cytoarchitectural and volumetric changes [13, 14]. Glutamatergic abnormalities have additionally been identified in plasma [15, 16] and CSF in MDD patients [17, 18]. Structural and functional alterations of GABAergic circuits have been shown to contribute to the pathophysiology of anxiety and MDD [19, 20]. Reduced GABA levels in anterior cingulate cortex (ACC) in patients with MDD have been observed using magnetic resonance spectroscopy imaging [12, 21]. A downregulation of several GABA related genes in subgenual ACC (sgACC) has also been reported in patients with MDD, demonstrating altered GABAergic circuitry in key regions associated with MDD [22]. Reduced GABA levels have also been shown in plasma [23] and the CSF [24] in patients with MDD, reflecting decreased levels of extracellular GABA in the brain or decreased turnover.

The majority of cortical neurons and synapses use glutamate as a neurotransmitter, therefore it has been suggested that the glutamatergic system is a primary mediator of depression, and may represent a common pathway for the therapeutic action of certain antidepressant compounds [9]. Disrupted excitatory and inhibitory (E/I) neurotransmission is thought to contribute greatly to the development of mood disorders [21], possibly through impaired synaptic function [25, 26]. The sgACC is a key region in mood regulation, as well as in the symptomatic expression of depression, displaying structural, functional and metabolic abnormalities [27, 28].

Abnormalities in glutamatergic and GABAergic neurotransmission in the sgACC in MDD are well established [22, 29, 30], but how these changes relate to depression in DLB is unknown. In DLB, MR spectroscopy approaches have indicated changes in glutamate based on lower occipital cortex glutamate and N-acetyl aspartate, lower than observed in Alzheimer’s disease [31]. Similarly, reduced glutamate/glutamine to creatine ratios are seen in multiple regions in DLB and are potentially greater than in Alzheimer’s disease [32]. In Parkinson’s disease with depression, elevated GABA signals in the frontal cortex correlate with the presence of depression [33], suggesting an imbalance in glutamate/GABA signalling specifically in relation to depression.

With these changes of glutamate and GABA in neurodegeneration and associations with depression, we assessed GABAergic and glutamatergic synaptic function and neurochemical changes in relation to depression in DLB. We additionally used data from previous studies to determine interactions between dopaminergic and serotonergic transmission that contribute to depression in DLB [8].

## Methods

### Post-mortem human brain tissue

Post-mortem human brain tissue was obtained from the Newcastle Brain Tissue Resource, with ethical approval granted by Newcastle and North Tyneside National Health Service Research Ethics Committee (ref: 09/H0908/42). All participants had received detailed clinical assessments during their life and had consented to the use of their brain tissue for research purposes. Neuropathological assessment was carried out according to standardised neuropathological diagnostic procedures. All cases were as closely matched as possible for age, sex and post-mortem delay. The inclusion criteria for depression diagnosis was made using the Cornell Scale for Depression in Dementia (≥8), as a validated rating scale for depression in dementia [34]. Alternatively, the Geriatric Depression Scale (≥10) was used, which shows acceptable qualities when applied to a population of demented elderly patients [35]. In the absence of clinical diagnosis of depression by a consulting psychiatrist, retrospective analysis of clinical records was used to verify the presence or absence of depression. For confocal and adapted stereological analysis 17 normal controls, 17 DLB cases without depression and 15 DLB cases with depression were used (see Supplementary Table 1). The cohort for biochemical analysis consisted of 12 normal controls, 12 DLB cases without depression and 12 DLB cases with depression (see Supplementary Table 2). Four DLB cases also fulfilled the neuropathological criteria for high AD neuropathological change and could therefore be classified as neuropathologically mixed AD/DLB with a Lewy body disease (LBD) clinical phenotype [36].

### Immunohistochemistry

Formalin fixed paraffin embedded tissue blocks of the sgACC (Brodmann area: BA25) at the level of the rostrum of the corpus callosum were cut at 10μm and sections mounted onto charged glass slides (Superfrost; Thermo Scientific, UK). Following paraffin wax removal through xylene, the sections were rehydrated with decreasing concentrations of ethanol. Heat-induced antigen retrieval was carried out in 0.1 M trisodium citrate pH 6.0 buffer for 10 min. For adapted stereological analysis of neuronal density, sections were incubated in 3% hydrogen peroxide, followed by two washes in 10 mM tris-buffered saline (TBS) containing 0.1% v/v Tween 20 (TBST; pH 7.6). Tissue sections were incubated with primary antibodies (see Supplementary Table 3) diluted in TBST for one hour at room temperature, then incubated with horseradish peroxidase (HRP) polymer conjugated universal probe (MenaPath, Menarini Diagnostics UK) for 30 min, followed by incubation with HRP reagent for 30 min, with three TBST washes in-between. Visualisation of the antibody was attained by using the Menarini X-Cell-Plus HRP Detection Kit (Menarini, UK), using Diaminobenzidine (DAB) substrate for two minutes. Sections were counterstained with Mayer’s haematoxylin, dehydrated and mounted using DPX resin (Cell Path, UK). An adapted, previously described, stereological method was used to estimate the number of GABAergic (GAD-67) and glutamatergic (CRYM) neurones in the sgACC [37].

For confocal analysis of glutamatergic and GABAergic synapses in the sgACC, quadruple immunofluorescence was used to assess the number and volume of synapses in relation to phosphorylated α-synuclein (s129). Following heat-induced antigen retrieval, sections were blocked for one hour at room temperature in TBS containing 10% normal goat serum (Sigma, UK) to prevent non-specific binding, which was also used to dilute primary and secondary antibodies. The tissue sections were incubated with primary antibodies overnight at 4°C. After three washes in TBS, sections were incubated with secondary fluorescent antibodies for 1 h at room temperature, followed by three washes in TBS. To stain the nuclei, sections were incubated with TO-PRO-3 Iodide (1 mM Solution in DMSO, Thermo Fisher, UK) for 15 min, followed by TBS washes. Prior to and following an incubation in Sudan Black B (0.3%w/v in 70% ethanol) for 10 min to quench autofluorescence, sections were immersed in 70% ethanol for 5 s, then washed in deionised water. Sections were mounted using ProLong Diamond Antifade Mountant (Thermo Fisher, UK) and stored at −30 °C to preserve the fluorescent signal prior to imaging.

### Confocal imaging and analysis

Fluorescent quadruple labelled sections were imaged using Nikon A1R confocal microscope and NiS elements software using 60X oil immersion objective [38]. The settings for laser power, gain and offset were optimised for each fluorophore (405 nm, 488 nm, 546 nm and 647 nm). Ti ZDrive construction of the Z-stacks was attained by determining the lower and upper thresholds of the section depth, with multiple images captured at 0.17μm intervals through the depth of the tissue. Image averaging was set to 16X to reduce background interference and provide an accurate depiction of the tissue stain. Four images were acquired per case within the region of interest. To analyse GABAergic and glutamatergic synaptic volumes in relation to α-synuclein, IMARIS software (Bitplane, Oxford Instruments) was used to generate 3D representative images from the imported Z-stacks, with individual surfaces created for each fluorescent signal. 3D surfaces were created for Nuclei (647 nm, purple), GAD65/67 positive GABAergic surfaces or VGLUT1 positive glutamatergic surfaces (488 nm, green), SNAP25 positive pre-synaptic terminals (546 nm, red) and s129 positive α-synuclein surfaces (405 nm, blue). The staining intensity boundaries were optimised for each laser channel (405 nm 500–3500, 488 nm 600–3500, 546 nm 400–3000), whereas background subtraction of 1.0 and smoothness of 0.1 was applied to all the channels. The number and the average volume of individual surfaces for different laser channels was calculated and averaged across four Z stacks. The number and average volume of GABAergic and glutamatergic synapses was determined based on the co-localisation between GAD65/67 or VGLUT1 positive surfaces and SNAP25. The synaptic surface was then masked onto α-synuclein, which allowed synapses containing α-synuclein to be selected, creating surfaces for the α-synuclein positive synapses.

### Protein analysis

Proteins involved in glutamatergic and GABAergic neurotransmission were quantified using dot blot analysis. Approximately 50 mg unfixed frozen grey matter corresponding to sgACC was homogenised using a rotor-stator homogeniser in ice cold lysis buffer consisting of 0.2 M triethylammonium bicarbonate pH 8.0 (Sigma-Aldrich, UK), and 1X EDTA free protease inhibitor cocktail (Complete, Roche, UK). Protein concentrations were determined using Bradford assay against standards of known protein concentration prepared using bovine serum albumin (Sigma-Aldrich, UK). Protein samples for dot blot were prepared at 1 µg/µl with 4X Orange G Loading Buffer, 10X NuPAGE Sample Reducing Agent (Invitrogen) and homogenising buffer. Tissue protein standards ranging from 0 μg/μl to 120 μg/μl were prepared using pooled samples from all disease groups. The samples were denatured at 70°C for 10 min on a heated block prior to loading. A vacuum-assisted 96-well dot blot apparatus (Hoefer Inc., USA) was used to blot the samples onto nitrocellulose membrane (Amersham^TM^, 0.2μm NC) pre-wetted with TBS. Fifty microliters of sample or standards were pipetted into each well in duplicate. The membrane was then washed with TBS (100 μl per well), left to dry on a flat surface for 15 min, fixed in 70% methanol for 20 min under agitation, rinsed in MilliQ water and washed in TBS. The membranes were blocked using Odyssey blocking buffer (LI-COR), then incubated overnight with primary antibodies (see Supplementary Table 3) diluted in Odyssey blocking buffer and 0.2% Tween20 (Sigma-Aldrich) at 4°C. Membranes were washed in TBST three times for 5 min at room temperature, then incubated with IRDye 800WC secondary antibodies diluted in Odyssey blocking buffer and 0.2% Tween20 for 1 h. Following three TBST washes, membranes were incubated with IRDye conjugated GAPDH 680 nm (Santa Cruz Biotechnology) as an internal loading control diluted in Odyssey blocking buffer and 0.2% Tween20 for 1 h at room temperature. Before imaging, membranes were washed three times in TBST for 5 min, followed by two 5 min washes with TBS to remove residual Tween20 and imaged while wet. Each membrane was scanned at 700 nm (GAPDH) and 800 nm (protein of interest) for 2 min. The protein intensity bands were quantified using Image Studio Lite (LI-COR). The Lane Normalisation Factor (LNF) was calculated by dividing GAPDH signal for each lane by the highest GAPDH signal. The signal for the protein of interest was then divided by the LNF to normalise the protein of interest.

### Statistical analyses

Statistical analyses were performed using SPSS Statistics version 22.0. The normal distribution across samples was assessed using Shapiro–Wilk test, with homogeneity of variance determined using Levene’s test. If the assumptions of normality were met, analysis of variance (ANOVA) was used to analyse the data sets between the groups, followed by Bonferroni post-hoc analysis to correct for multiple comparisons. A paired-samples t-test was used for pairwise comparisons within groups. Where normal distribution criteria was not fulfilled, a non-parametric Kruskal-Wallis test was used to compare multiple groups, with adjusted p-values to correct for multiple comparisons, so that the overall error rate remained at 5%. Based on sample size calculations using Cohen’s recommendations, a change of 30% could be detected with power of 0.96 at alpha=0.05 in a given sample with equal group sizes of 12 per group.

The range of variation in expressed proteins amongst DLB patients was investigated using a combination of constrained ordination techniques, correlation networks, regression and discriminant analysis. Prior protein data relating to serotonin and dopamine neurotransmission was also used to determine any association with GABA and glutamate [8]. Canonical correspondence analysis (CCA) was used to investigate the major trends in variation of expressed proteins amongst DLB patients with and without depression with a view to identifying the suite of proteins most closely associated with depression. We used correlation networks to investigate how the expression of the proteins are correlated with each other in depressed and non-depressed DLB cases, followed by the generalised linear modelling. Finally, we used linear discriminant analysis to identify proteins whose expression levels can be used to identify and discriminate between depressed and non-depressed patients. Analyses were undertaken in R using the vegan, klaR and MASS packages with plots constructed using the ggplot2 and core network packages.

## Results

### Glutamatergic and GABAergic neuronal counts

**Fig. 1:**
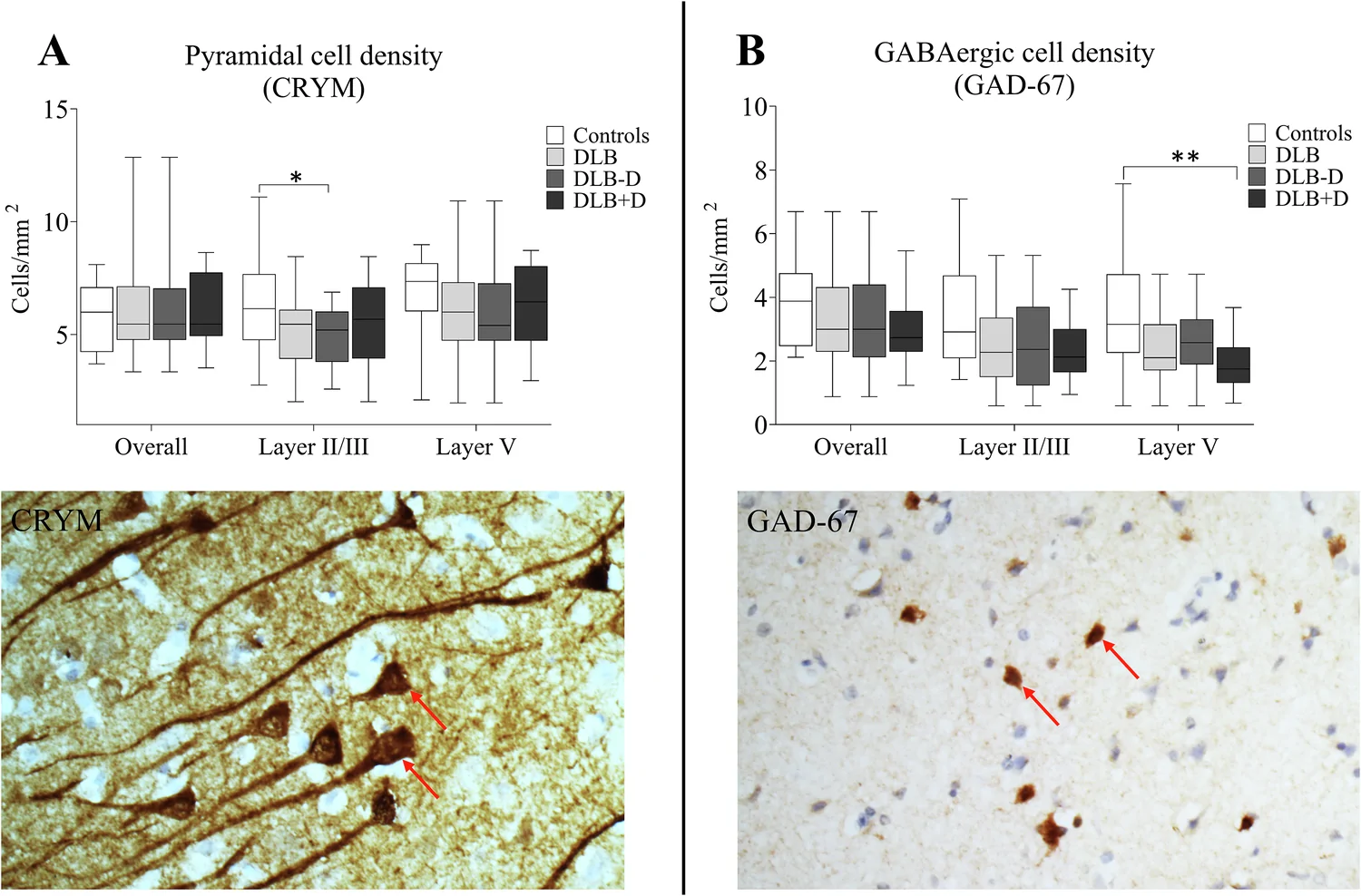
Glutamatergic and GABAergic cell density in sgACC. **(A)** CRYM positive pyramidal neurone and **(B)** GAD-67 positive GABAergic interneurone density was assessed in sgACC overall, as well as in layers II/III and layer V in controls (n = 17), DLB cases overall (n = 32), DLB cases without depression (DLB-D; n = 17) and DLB cases with depression (DLB + D; n = 15). The images were captured at 20X magnification; (*p < 0.05 and **p < 0.01).

Neuropathological changes did not associate with depression in DLB (Supplementary Tables 1, 2). To assess changes in excitatory and inhibitory cell populations within sgACC we used an adapted stereological method. Pyramidal neurones labelled with μ-crystallin (CRYM) were significantly reduced in layers II/III in DLB cases without depression compared to controls (*p* = 0.037; Fig. 1A), whereas GABAergic cells (GAD-67 positive) showed a significant decrease in layer V in DLB cases with depression compared to controls (*p* = 0.007; Fig. 1B).

### Synaptic changes in sgACC

**Fig. 2:**
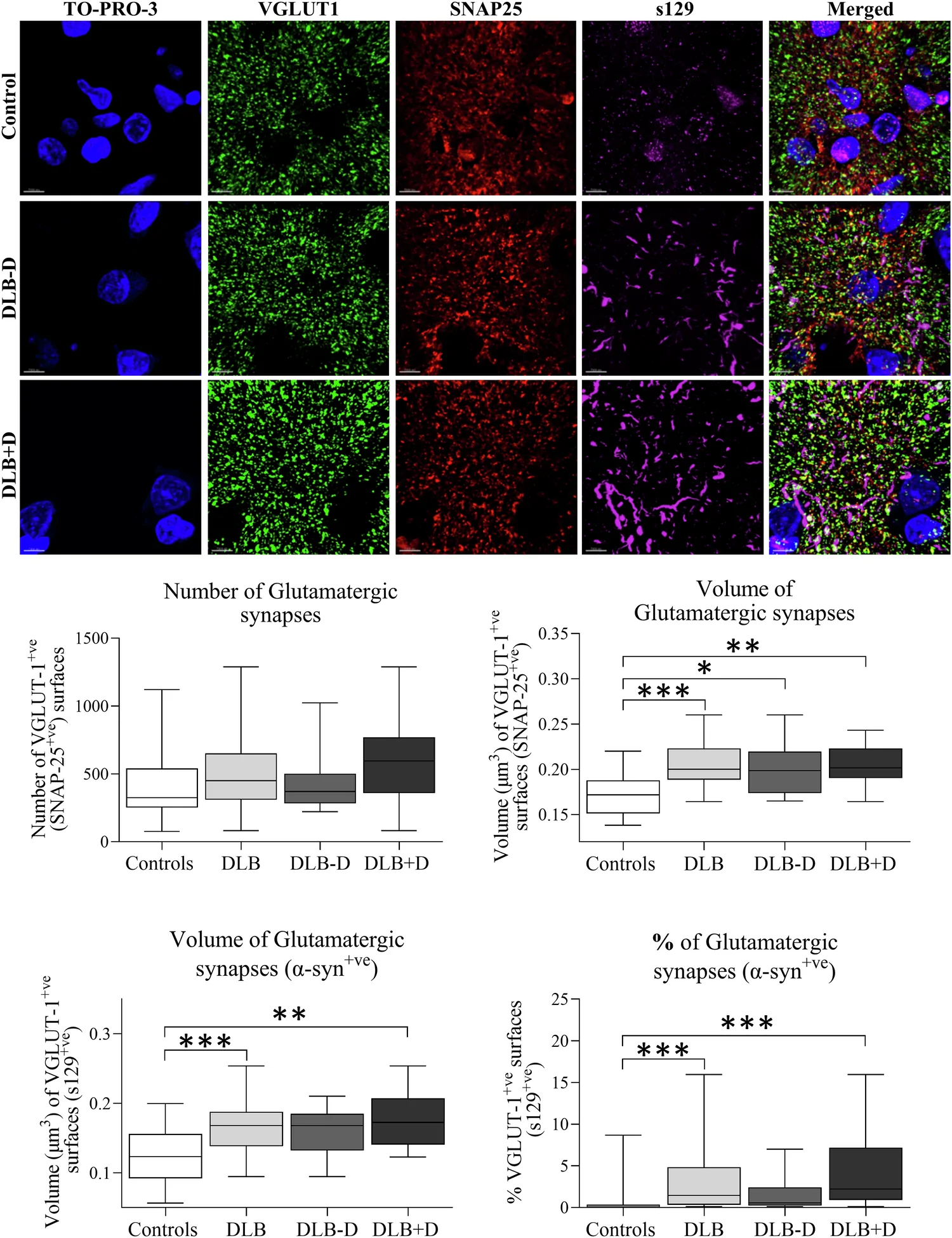
Confocal imaging and analysis of glutamatergic synapses in DLB. Representative images of glutamatergic synaptic immunoreactivity (VGLUT1 + SNAP25) in relation to s129 α-synuclein in the sgACC in age matched controls, DLB cases overall and DLB cases with (DLB + D) and without (DLB-D) depression. The nuclei were visualised using TO-PRO-3 Iodide (blue, 647 nm), glutamatergic synaptic terminals were visualised using VGLUT1 (green, 488 nm), presynaptic terminals demonstrated using SNAP25 (red, 546 nm) and α-synuclein immunoreactivity shown using s129 (purple, 405 nm). Scale bar represents 7μm. The number, volume and percentage of glutamatergic (VGLUT1 + SNAP25) presynaptic surfaces was assessed in sgACC in age matched controls, DLB cases overall, DLB cases with (DLB + D) and without (DLB-D) depression. (*, *p* < 0.05, **, *p* < 0.01, or *** *p* < 0.001, compared to control).

**Fig. 3:**
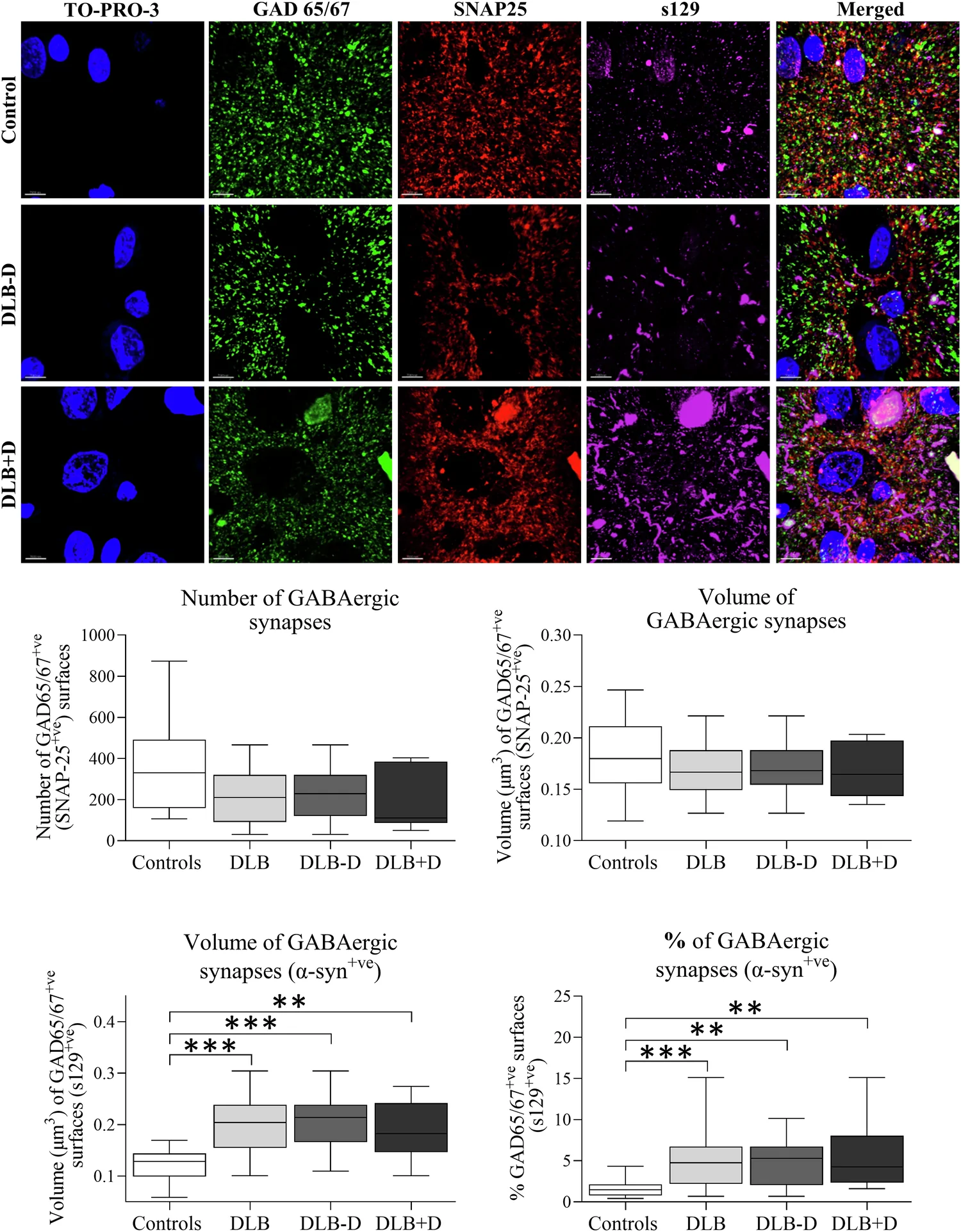
Confocal imaging and analysis of GABAergic synapses in DLB. Representative images of GABAergic synaptic immunoreactivity (GAD65/67 + SNAP25) in relation to s129 α-synuclein in the sgACC in age matched controls, DLB cases overall and DLB cases with (DLB + D) and without (DLB-D) depression. The nuclei were visualised using TO-PRO-3 Iodide (blue, 647 nm), GABAergic synaptic terminals were visualised using GAD 65/67 (green, 488 nm), presynaptic terminals identified using SNAP25 (red, 546 nm) and α-synuclein immunoreactivity using s129 (purple, 405 nm). Scale bar represents 7μm. The number, volume and percentage of GABAergic (GAD 65/67 + SNAP25) presynaptic surfaces was assessed in sgACC in age matched controls, DLB cases overall, DLB cases with (DLB + D) and without (DLB-D) depression. (*, *p* < 0.05, **, *p* < 0.01, or *** *p* < 0.001, compared to control).

Using confocal microscopy, the impact of α-synuclein pathology on glutamatergic (VGLUT1 and SNAP25 positive; Fig. 2) and GABAergic synapses (GAD65/67 and SNAP25 positive; Fig. 3) was assessed. Glutamatergic synapse numbers were not significantly different between the groups (*p* = 0.206). Glutamatergic synaptic volume was significantly higher in DLB cases overall (*p* = 0.003), and in DLB cases with (*p* = 0.003) or without depression (*p* = 0.017) compared to controls (Fig. 2). The volume of α-synuclein positive glutamatergic synapses was significantly higher in DLB cases overall (*p* = 0.004) and specifically in DLB cases with depression compared to controls (*p* = 0.002; Fig. 2). The proportion of glutamatergic synapses containing s129 α-synuclein was significantly higher in DLB cases overall (*p* = 0.011) and DLB cases with depression compared to controls (*p* = 0.001; Fig. 2).

The number (*p* = 0.304) and volume (*p* = 0.546) of GABAergic presynaptic surfaces was not significantly different between the groups (Fig. 3). The volume of GABAergic synapses containing 129 α-synuclein was significantly higher in DLB cases overall (*p* < 0.001), in DLB cases with (*p* = 0.003) and without depression (*p* < 0.001) compared to controls (Fig. 3). The percentage of GABAergic synapses containing 129 α-synuclein was also significantly higher in DLB cases overall (*p* = 0.007), DLB cases with (*p* = 0.028) and without depression compared to controls (*p* = 0.040; Fig. 3).

### Neurochemical changes associated with excitatory and inhibitory function

**Fig. 4:**
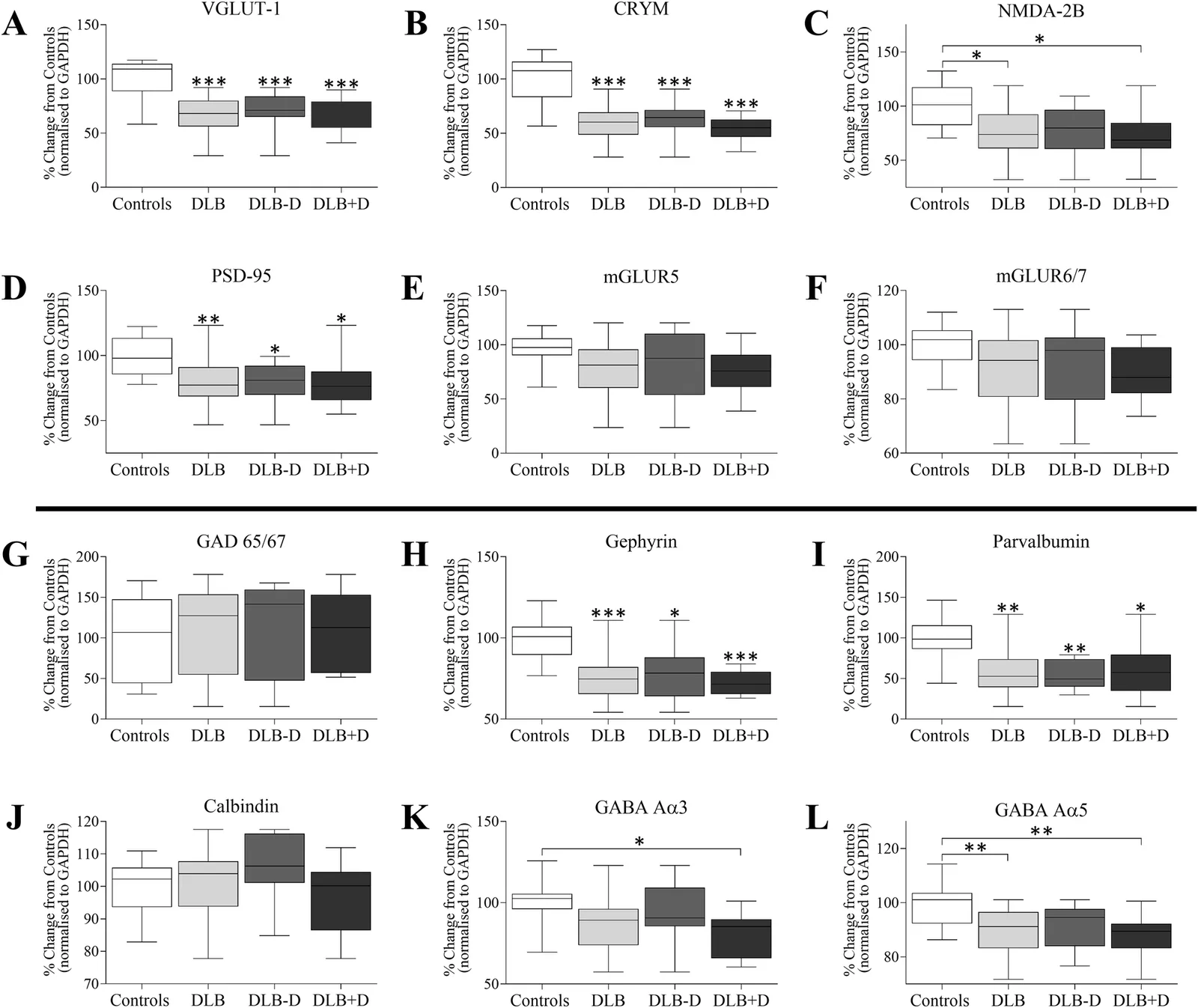
Protein analysis of glutamatergic and GABAergic markers in DLB. (**A**) VGLUT-1, (**B**) CRYM, (**C**) NMDA-2B, (**D**) PSD-95, (**E**) mGLUR5; (**F**) mGLUR6/7; (**G**) GAD-65/67, (**H**) Gephyrin, (**I**) Parvalbumin, (**J**) Calbindin, (**K**) GABA_A_α3 and (**L**) GABA_A_α5 protein concentrations were assessed in sgACC in age matched controls (n = 12), DLB cases overall (n = 24), DLB cases with (DLB + D; n = 12) and without (DLB-D; n = 12) depression. (*, *p* < 0.05 and ***, *p* < 0.001 compared to control). Box plots represent the median (midline) and quartile ranges, with error bars representing the minimum and maximum values.

To assess glutamatergic and GABAergic alterations on depression in DLB, specific protein levels in the sgACC were analysed. Vesicular glutamate uptake (VGLUT-1) was significantly lower in DLB cases overall, as well as DLB cases with or without depression compared to controls (*p* < 0.001; Fig. 4A). CRYM protein levels as a marker of glutamatergic neurones in sgACC were significantly lower in DLB cases overall, DLB cases with and without depression compared to controls (*p* < 0.001; Fig. 4B). NMDA receptor 2B subunit protein levels in sgACC showed significant decrease in DLB cases overall (*p* = 0.024) and DLB cases with depression compared to controls (*p* = 0.031; Fig. 4C). PSD95, involved in postsynaptic glutamate receptor clustering was significantly reduced in DLB cases overall (*p* = 0.004), and in DLB cases with (*p* = 0.011) and without depression (*p* = 0.022) (Fig. 4D). Metabotropic glutamate receptor levels in sgACC, including mGLUR5 (*p* = 0.187) and mGLUR6/7 (*p* = 0.151), were not significantly different between the groups (Fig. 4E, F).

No significant changes in GAD-65/67 protein levels in sgACC were detected between the groups (*p* = 0.777; Fig. 4G). Gephyrin, involved in GABA A receptor clustering of at inhibitory synapses, showed a significant decrease in DLB cases overall and DLB cases with depression (*p* < 0.001), and in DLB cases without depression (*p* = 0.015; Fig. 4H). Selective GABAergic protein changes were observed, including parvalbumin, which showed reduced levels in DLB cases overall (*p* = 0.002), DLB cases with (*p* = 0.021), and without depression compared to controls (*p* = 0.006; Fig. 4I). No significant changes were observed in calbindin protein levels between the groups (*p* = 0.110; Fig. 4J). A significant reduction was seen in GABA_A_ receptor α3 subunit in DLB cases with depression compared to controls (*p* = 0.015; Fig. 4K), and in GABA_A_ receptor α5 subunit protein levels, with significant decrease observed in DLB cases overall (*p* = 0.004) and DLB cases with depression compared to controls (*p* = 0.002; Fig. 4L).

**Fig. 5:**
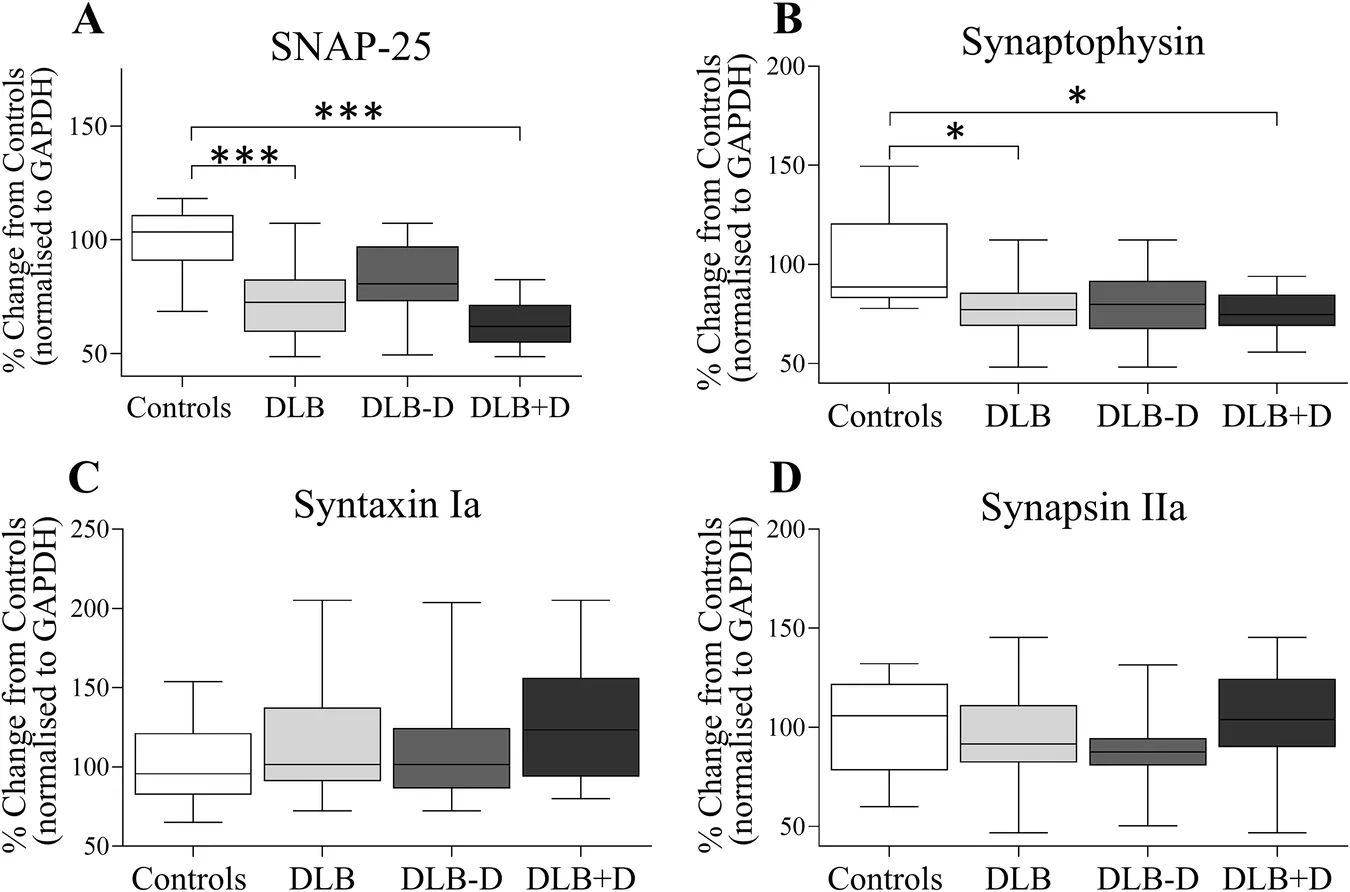
Protein analysis of synaptic markers in DLB. (**A**) SNAP-25, (**B**) Synaptophysin, (**C**) Syntaxin Ia, (**D**) and Synapsin IIa, protein concentrations were assessed in sgACC in age matched controls (n = 12), DLB cases overall (n = 24), DLB cases with (DLB + D; n = 12) and without (DLB-D; n = 12) depression. (*p < 0.05 and ***p < 0.001 compared to controls). Box plots represent the median (midline) and quartile ranges, with error bars representing the minimum and maximum values.

The synaptic marker protein SNAP25 was significantly different between groups (*p* < 0.001), with a significant decrease observed in DLB cases with depression (*p* < 0.001) and DLB cases overall (*p* < 0.001) compared to controls (Fig. 5A). Synaptophysin levels in the sgACC were significantly different between groups (*p* = 0.014), with a significant decrease observed in DLB cases overall (*p* = 0.023) and DLB cases with depression compared to controls (*p* = 0.025; Fig. 5B). Syntaxin Ia (*p* = 0.487) and Synapsin IIa (*p* = 0.426) protein levels showed no significant changes between the groups (Fig. 5C, D).

### Correlation networks and regression analyses

**Fig. 6:**
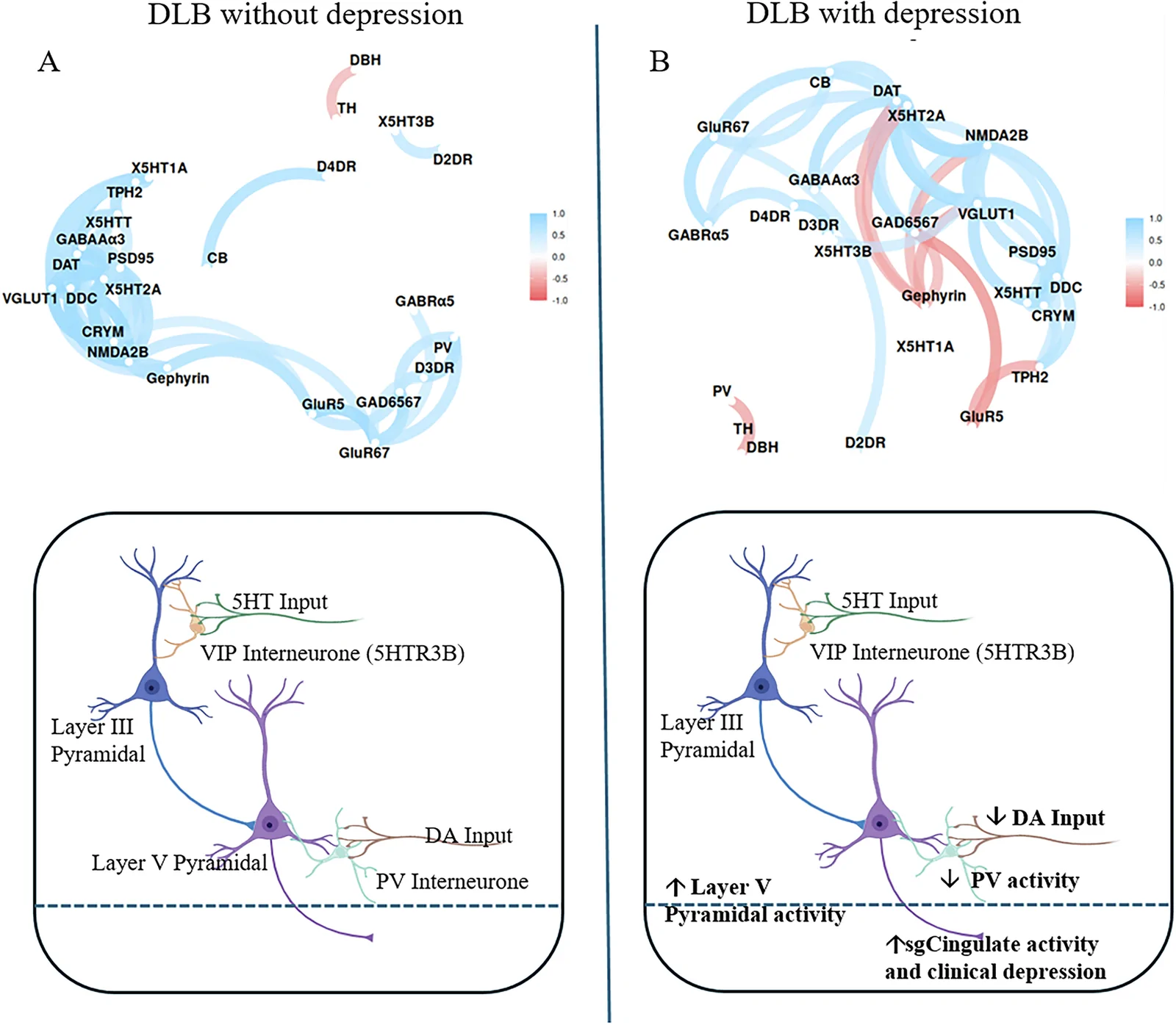
Correlation networks of protein levels for depressed and non-depressed cases. The correlation networks of non-depressed (**A**) and depressed (**B**) cases. Significant correlations (critical value at 5% = 0.57) are shown. The results indicate that there were significant negative correlations between GluR5 and TPH2; GluR5 and GAD6567; Gephyrin and X5HT2A as well as GAD6567 and NMDA2B that were not present in non-depressed patients.

As dopamine and serotonin play significant roles in mediating depression, we utilised existing data [8] to determine interactions with glutamate and GABA. The correlation networks of depressed and non-depressed cases were different (Fig. 6) with a closely constrained network in non-depressed donors and an open network in depressed DLB donors. The results indicate that there were significant negative correlations between GluR5 and TPH2, GluR5 and GAD65/67, Gephyrin and 5HT2A as well as GAD6567 and NMDA2B that were not present in non-depressed patients. However, the analyses of correlation networks indicates that the system is more complex, as there were clear negative associations between five proteins in depressed cases (GluR5, NMDA2B, Gephyrin, GAD65/67, and TPH2) that did not occur in non-depressed cases. This shows that multivariate approaches that investigate the variation across all of the variables collectively might provide a better understanding than the initial univariate regression analyses reported above.

### Multivariate analyses

A constrained ordination (CCA: see supplementary figure 1) showed 5HTT and PV were most expressed in depressed patients whilst 5HT3B was expressed more in patients without depression. The greedy.wilks algorithm was used to investigate the extent to which depressed and non-depressed patients could be distinguished on the basis of the levels of the proteins measured across the two groups. Two models were identified that led to high levels of discrimination. Levels of 5HT3B and CB led to 83.3% correct allocation to depressed/non-depressed state in a reclassification of the original data. The discriminatory power of 5HT3B is mirrored in the CCA where high levels of 5HT3B were associated with the absence of depression. Relationships between level of protein expression and depression status using regression analysis showed four proteins, CB, D4DR, D3DR, and 5HT3B, with significant differences between depressed and non-depressed cases. This regression analyses supported the utility of 5HT3B as a discriminator.

## Discussion

Depression is an important clinical feature in many people with DLB and has a major impact on the quality of life of both patients and carers [4–6]. Given the lack of effective treatment options, we have explored the aetiology of depression in DLB using post-mortem tissue. Neuropathological changes do not appear to associate with depression in DLB [8] and in this study we saw no difference in α-synuclein or tau staging between depressed and non-depressed groups (Supplementary Table 1, 2). Previous studies have however, shown dopaminergic changes to correlate with depression in DLB [8], however given the complex aetiology, dopamine may not define all aspects of depression. Herein, we investigated glutamatergic and GABAergic changes in the sgACC in DLB, focussing on the sgACC as a region implicated in depression and showing significant pathology in DLB. Our findings suggest that an imbalance in excitatory and inhibitory neurotransmission may contribute to depression in DLB. Furthermore, neurotransmitter receptor and synaptic protein changes in the sgACC may contribute to synaptic dysfunction in DLB, possibly due to accumulation of aggregated α-synuclein within presynaptic terminals.

Whilst we observed no overall changes in neuronal cell number, mild pyramidal cell loss was observed in superficial layers of the sgACC in DLB cases without depression. Although changes in the overall number of glutamatergic synapses were not seen using confocal microscopy, reductions in CRYM and VGLUT1 protein levels in sgACC were observed in DLB, suggesting functional pyramidal cell abnormalities. VGLUT levels are critical in determining the balance between excitation and inhibition [39], and are also implicated in mood disorders, with reduced VLGUT1 expression in the hippocampus observed in animal models [40, 41] and in human studies of depression [42]. Loss of glutamatergic synapses in Alzheimer’s disease occurs [43] and in the parietal and occipital cortex this correlates with the presence of depression [44]. Our findings indicate that glutamatergic changes are seen in DLB corresponding to reduced NMDA 2B receptor expression, noticeably in DLB cases with depression. This accords with studies demonstrating a significant reduction in NMDA 2A and 2B subunit expression in the PFC of patients with MDD [26, 45] and metabotropic glutamate receptor (mGluR) reductions in animal models [46, 47]. Studies in Alzheimer’s disease have shown benefits in improving apathy with the use of the NMDA-receptor antagonist and dopamine D2 receptor agonist memantine [48] in some but not all studies [49]. In DLB, use of memantine to treat cognition has shown improved scores using the Neuropsychiatric Inventory (NPI) but not specifically with depression or anxiety [50], although in other studies no change in NPI has been seen [51]. Since most DLB patients show hallucinations as a core feature that may also relate to dopaminergic and glutamatergic changes [52], improvements in delusions and hallucinations observed with memantine may indicate an E/I imbalance in DLB also relates to hallucinations and warrants further study of these clinical features [50]. Larger scale trials with NMDA targeting compounds may be necessary to show useful benefit in treating depression, but potentially also hallucinations and delusions in DLB.

A decrease in layer V GABAergic neurones was observed in DLB cases with depression compared to controls, suggesting reduced inhibition of layer V pyramidal neurons and an increase or imbalance in excitation [53], possibly related to the presence of α-synuclein pathology which preferentially affects layer V/VI in DLB [54]. PV expressing GABAergic neurones constitute the largest group of layer V interneurons, potentially indicating selective vulnerability of specific interneurone populations. We observed no significant disease or depression specific changes in GAD65/67 protein levels in sgACC, whereas PV levels were significantly reduced in DLB cases, correlating with the reduction of layer V interneurones and suggesting selective PV interneurone changes. While some studies have observed reduced GAD67 protein and gene expression in the DLPFC and sgACC in MDD [29, 55], others found no changes in GAD65 or GAD67 cell numbers and mRNA levels in DLPFC in MDD [56]. Decreased PV gene expression was observed in sgACC post-mortem tissues in MDD [29], with PV interneurones shown to contribute to regulation of E/I within the PFC, influencing emotional responses [56]. Decreased expression of GABA_A_ receptor α3 subunit genes and increased expression of α5 has been observed in MDD [57, 58], suggesting distinct roles of GABA_A_ receptor subunits in MDD aetiology. Our findings demonstrated a significant reduction in GABA_A_ α5 and GABA_A_ α3 receptor subunits in DLB, particularly in DLB cases with depression compared to controls and this, accompanying the loss of PV interneurones, may underpin certain aspects of depression in DLB.

Changes in postsynaptic proteins involved in GABAergic (gephyrin) and glutamatergic (PSD95) neurotransmission have been associated with many neuropsychiatric disorders [59, 60], but in this study we observed disease, but not depression specific changes, with reduced PSD95 and gephyrin protein levels in DLB. Downregulation of glutamatergic and GABAergic receptors is often observed in MDD [26, 57, 58]. Synaptic abnormalities in DLB are suggested to precede neuronal loss and LB formation [61] and are closely related to clinical symptoms [62]. Phosphorylated α-synuclein (s129) has been detected in synaptic-enriched fractions in post-mortem DLB cases [63, 64], with synaptic α-synuclein toxicity observed in animal models [65, 66]. Studies in DLB have shown low expression of α-synuclein in inhibitory interneurons [67], sparing of LB pathology in cortical PV interneurones [68] and an absence of LB in hippocampal GAD positive neurones [69]. In this study, we observed the presence of s129 in both glutamatergic and GABAergic synapses. Synaptic dysfunction within the sgACC is also supported by our observation of unchanged numbers of GABAergic or glutamatergic synapses, but with an accompanying increase in the average volume of glutamatergic synapses in DLB. A significant increase in the volume of glutamatergic synapses containing s129 was found in DLB cases overall and DLB cases with depression compared to controls, whereas GABAergic synapses containing s129 were elevated in all DLB groups, although showing no selective depression specific changes. Our results are comparable with other studies, showing significantly increased size of glutamatergic synapses and presynaptic terminals containing s129 [70, 71]. This increased size may be a result of a compensatory response to the accumulation of pathological s129, however other compensatory mechanisms such as hypertrophy to increase declining synaptic activity may occur [38].

Alpha-synuclein directly interacts with SNARE complex proteins [72], essential in vesicle fusion and exocytosis [73]. SNARE complex dysfunction can result in presynaptic accumulation of endogenous α-synuclein, possibly representing the initial pathological event in DLB, and eventually leading to cellular dysfunction and death [70]. Abnormalities in SNAP25 and syntaxin expression have been shown to be involved in a wide range of neuropsychiatric disorders [74]. SNAP25 protein level was significantly reduced in DLB cases generally, and specifically in depressed DLB cases, with similar observations made in synaptophysin protein levels. These results suggest that both the machinery involved in synaptic vesicle exocytosis, and presynaptic proteins involved in efficient neurotransmitter release are altered, and accumulation of synaptic α-synuclein pathology may play a role in the pathogenesis of depression in DLB.

Our findings indicate that glutamatergic and GABAergic changes may play a role in the high rates of depression seen in DLB patients, and potentially also high rates of hallucinations. Advances in the development of fast-acting antidepressants has resulted in the development of selective glutamatergic and GABAergic agents. Esketamine, a non-competitive NMDA receptor antagonist is approved for use in treatment resistant MDD [75], and considering the successful use of the NMDA antagonist memantine to treat hallucinations in DLB, the use of esketamine has potential for treating multiple psychiatric features in DLB. Selective targeting of the NMDA 2B receptor using the antagonists MK-0657 and CP-101606, has also been shown to be effective in treating depression without serious side effects [76, 77]. Selective agonists or positive modulators of GABA_A_α3 subunit including TPA023 and eszopiclone have been proposed as potential antidepressants although mild sedation may mean that use in the elderly should be considered with caution [78, 79]. When co-administered with SSRIs, the preferential GABA_A_ α3 positive modulator eszopiclone induced a faster onset of efficacy and greater treatment response [80]. Preclinical studies showed that both positive and negative allosteric modulators of GABA_A_ α5 receptors (e.g. zonisamide) can also produce rapid antidepressant-like effects [81, 82]. Therefore, selective targeting of specific glutamatergic and GABAergic receptors might provide more effective treatment of depression and additionally hallucinations in DLB where there is a high and unmet medical need.

Since cortical circuits are modulated by subcortical projections and our previous study showed monoamine changes in DLB [8], we determined if any interaction occurred between dopaminergic and serotonergic systems and cortical glutamatergic and GABAergic neurones. Correlation network analysis (Fig. 6) showed tightly correlated circuits in non-depressed DLB donors but with diffuse correlation in the depressed DLB donors, where GluR5, GAD6567, NMDA2B, Gephyrin, TPH2 and 5HT2A showed negative correlations along with PV, DBH, and TH. Canonical correspondence analysis (Supplementary Figure 1) indicated that depressed DLB donors were more likely to show changes in PV and 5HT transporter (5HTT), whereas 5HT3B and CB defined the non-depressed DLB group with over 80% discrimination. This was observed using regression analysis (Supplementary Table 4) with CB and 5HT3B showing expression differences in non-depressed DLB, and in dopamine D3 and D4 receptors in depressed DLB donors as seen previously (Supplementary Table 4). This multivariate approach points towards a more subtle interaction between subcortical projections and cortical activity where serotonin and dopamine play a role in modifying cortical activity and particularly the E/I balance. Serotonin 5HT3B receptors are predominantly expressed in limbic brain regions [83], where 5HT3B receptors are typically expressed on GABAergic interneurones, and predominantly on upper layer II/III Vasoactive Intestinal Polypeptide (VIP) and 5HT3A receptor expressing interneurons [84, 85]. Activation of these 5HT3B receptors leads to enhanced GABA network activity and enhanced excitability [86, 87]. The possible imbalance in serotonergic input to cortical interneurones in depression in DLB may lead to abnormal activation of upper layer pyramidal neurones [88, 89] with preservation of 5HT input and 5HT3B receptors and relatively normal E/I balance in non-depressed DLB donors [8]. In contrast, lower layer PV interneurones co-express CB and are activated predominantly through dopamine [90, 91] with dopamine-dependent activation of PV interneurones leading to enhanced interneurone activity and reduced pyramidal neurone firing rates particularly in lower layer neurones [90, 92]. This combination of upper layer VIP/5HT3B hyperactivity and lower layer PV/DRD1/3 hypoactivity may lead to the abnormal sgACC hyperactivity and associated depression related states [93, 94]. Normalising the activity of the sgACC may potentially be achieved by reducing VIP interneurone activity and increasing PV interneurone activity. 5HT3 receptor antagonists such as ondansetron or the antidepressant vortioxetine which acts partly as a 5HT3 antagonist, have shown some beneficial effects in the treatment of depression in other disorders [95, 96]. Positive effects of dopamine agonists in depression and in PD may have their effects through PV interneurone function in reducing depressive symptoms [97, 98]. Use of the GABA_A_ α3 positive modulator eszopiclone combined with 5HT3 antagonist/dopamine agonist treatment may be beneficial in treating depression in DLB [80, 99].

In conclusion, DLB patients with depression show deficits in GABAergic and glutamatergic neurotransmission in the sgACC, potentially due to altered synaptic α-synuclein accumulation. This may lead to a greater imbalance in excitation and inhibition in depressed DLB donors compared to DLB cases without depression. Whether other regions involved in mediating or linked to depression such as the DLPFC, nucleus accumbens, or medio-dorsal thalamus [100] are involved, remains to be elucidated. Modifying GABAergic and glutamatergic function, however, may be a potential treatment strategy for depression in DLB.

## Supplementary information

Supplementary Material

## Data Availability

All data will be made available upon reasonable request to the authors.
