# Supplementary material for: Imbalance in excitation and inhibition in sgACC is associated with depression in dementia with Lewy bodies
Source: Transl Psychiatry. 2026 Jul 3;16:493. doi: 10.1038/s41398-026-04224-x (PMC13616906; doi:10.1038/s41398-026-04224-x)
Supplement: Supplementary file 1 [file 41398_2026_4224_MOESM1_ESM.docx]

**Supplementary Tables and Figures**

**Supplementary Table 1.** Demographic Information for Immunohistochemical Cohort

Controls (n=17), DLB without depression (DLB-D, n=17), DLB with depression (DLB+D, n=15); Age at death (ANOVA) *F*(2)=2.628, *p*=0.083; Post-mortem Interval (PMI, hours, Kruskal-Wallis) *H*(2)=0.506, *p*=0.777; Gender (Chi-squared test) χ2(2)=2.362, *p*=0.364; NFT Braak stage between DLB-D and DLB+D χ2(2)=0.53, *p*=0.974.

**Supplementary Table 2.** Demographic Information for Biochemical Cohort

Controls (n=12), DLB without depression (DLB-D, n=12), DLB with depression (DLB+D n=12); Age at death (ANOVA) *F*(2)=0.124, *p*=0.884; pH (ANOVA) *F*(2)=0.216, *p*=0.326; Post-mortem Interval (PMI, hours, Kruskal-Wallis) *H*(2)=6.190, *p*=0.045*, Gender (Chi-squared test) χ2(2)=1.636, *p*=0.576; NFT Braak stage between DLB-D and DLB+D χ2(2)=0.48, *p*=0.977. * PMI not significant between groups after post-hoc analysis.

**Supplementary Table 3.** Antibodies used in the study.

DB – dot blot; F-IHC – fluorescent immunohistochemistry.

**Supplementary Figure 1.** **Multivariate analyses.**

Constrained Ordination (CCA) plot of protein expression scores for individual proteins in depressed and non-depressed DLB patients. Red points represent the score for the depressed and non-depressed state.
